# Supplementary material for: Structural basis of FPR2 in recognition of Aβ42 and neuroprotection by humanin
Source: Nat Commun. 2022 Apr 1;13:1775. doi: 10.1038/s41467-022-29361-x (PMC8976073; doi:10.1038/s41467-022-29361-x)
Supplement: Supplementary file 1 — Supplementary Information [file 41467_2022_29361_MOESM1_ESM.pdf]

Supplementary Information

for

Structural basis of FPR2 in recognition of Aβ_{42} and neuroprotection by humanin

Ya Zhu, Xiaowen Lin, et al.

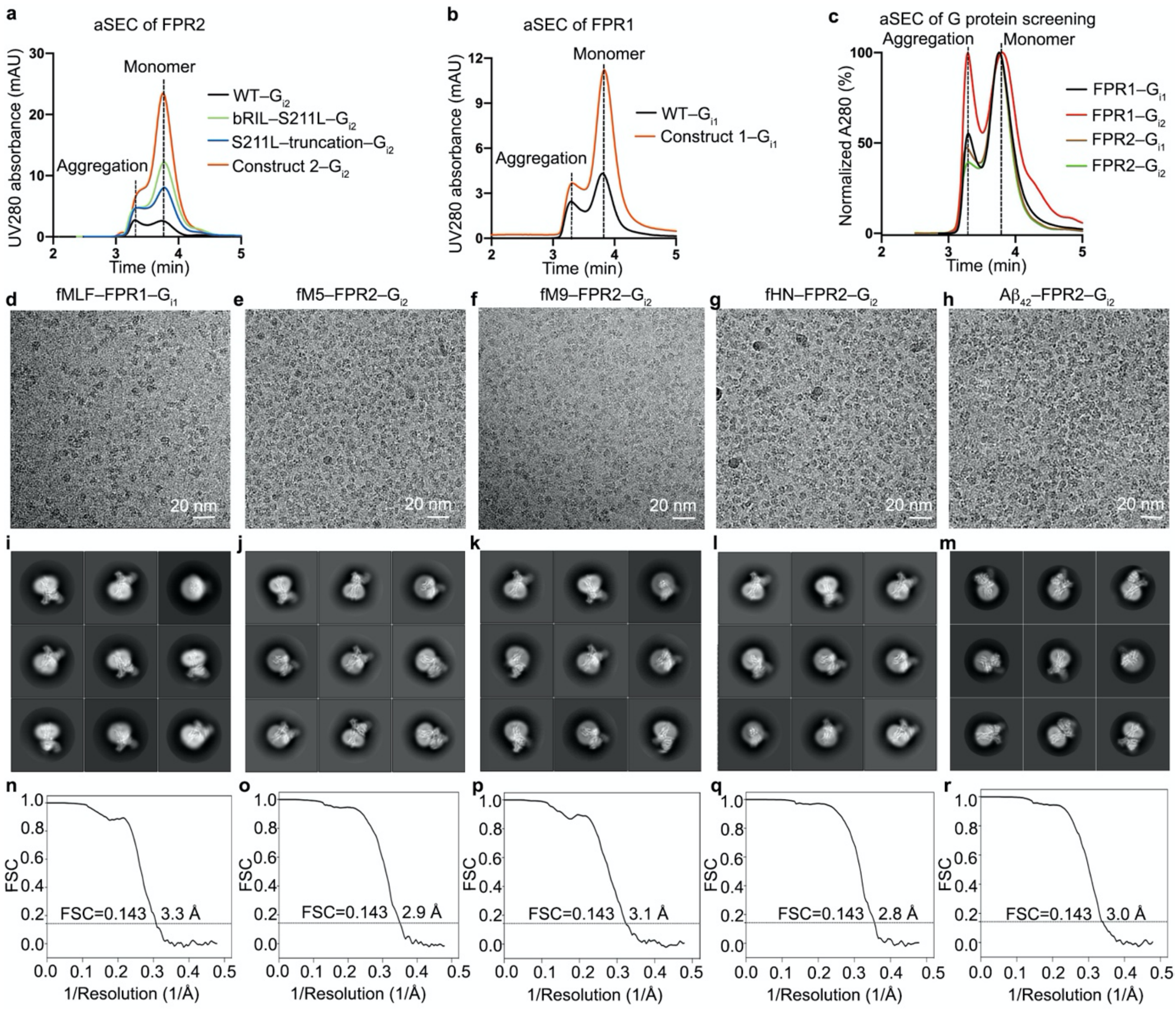

Supplementary Figure 1. Sample preparation and cryo-EM of FPR1 and FPR2 complexes. a, Analytical size-exclusion chromatography (aSEC) of wild-type FPR2 (WT) and modified constructs in complex with fHN and G_{i2} using a 4.6 × 250 mm Nanofilm SEC-250 column (Sepax Technologies). Construct 2 is the FPR2 construct used to determine the peptide agonist–FPR2–G_{i2} structures. The receptor in monomeric and aggregational forms are indicated by two dashed lines. The data show that the mutation S211$^{5.48}$L, bRIL fusion and C-terminal truncation of FPR2 improved protein yield and homogeneity of the G_{i2}-bound complex. **b**, aSEC of wild-type FPR1 (WT) and construct 1 in complex with fMLF and G_{i1}. Construct 1 is the FPR1 construct used to determine the fMLF–FPR1–G_{i1} structure. The data show that the C-terminal truncation of FPR1 improved protein yield and homogeneity of the G_{i1}-bound complex. **c**, aSEC of FPR1–fMLF and FPR2–fHN in complex with G_{i1} or G_{i2}. The

data show that fMLF–FPR1–G_{i1} and fHN–FPR2–G_{i2} have better homogeneity than fMLF–FPR1–G_{i2} and fHN–FPR2–G_{i1}, respectively. **d-h**, Representative cryo-EM images of fMLF–FPR1–G_{i1}, fM5–FPR2–G_{i2}, fM9–FPR2–G_{i2}, fHN-FPR2–G_{i2} and $A\beta_{42}$–FPR2–G_{i2} complexes from three independent experiments with similar results. **i-m**, Two-dimensional averages of fMLF–FPR1–G_{i1}, fM5–FPR2–G_{i2}, fM9–FPR2–G_{i2}, fHN-FPR2–G_{i2} and $A\beta_{42}$–FPR2–G_{i2} complexes. **n-r**, Gold-standard Fourier shell correlation (FSC) curves of fMLF–FPR1–G_{i1}, fM5–FPR2–G_{i2}, fM9–FPR2–G_{i2}, fHN-FPR2–G_{i2} and $A\beta_{42}$–FPR2–G_{i2} complexes.

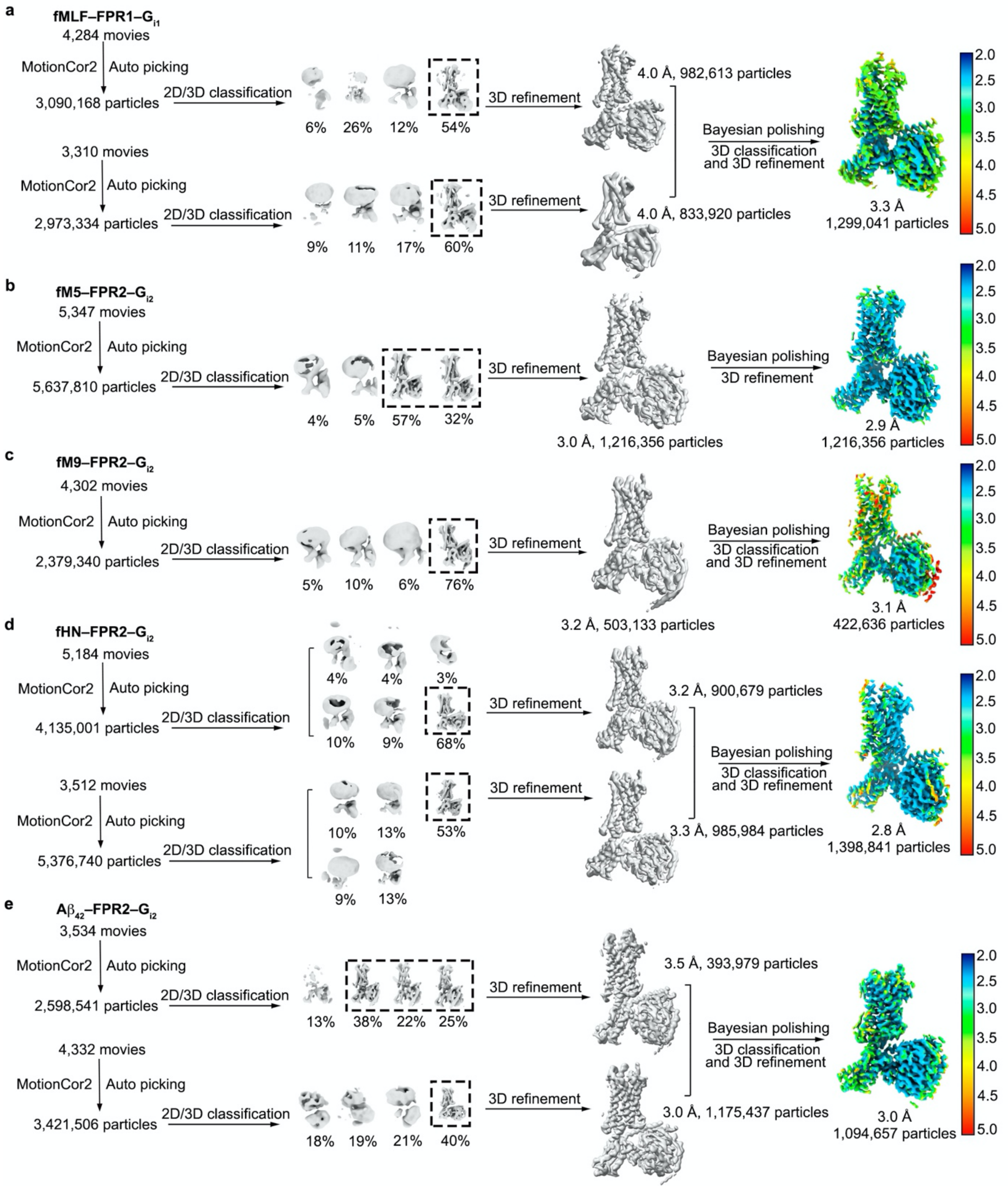

Supplementary Figure 2. Workflow of cryo-EM data processing with cryo-EM map colored according to local resolution (Å). a, fMLF–FPR1–G_{i1}; **b**, fM5–FPR2–G_{i2}; **c**, fM9–FPR2–G_{i2}; **d**, fHN–FPR2–G_{i2}; **e**, $A\beta_{42}$–FPR2–G_{i2}. The dashed boxes indicate the classes used for 3D refinement.

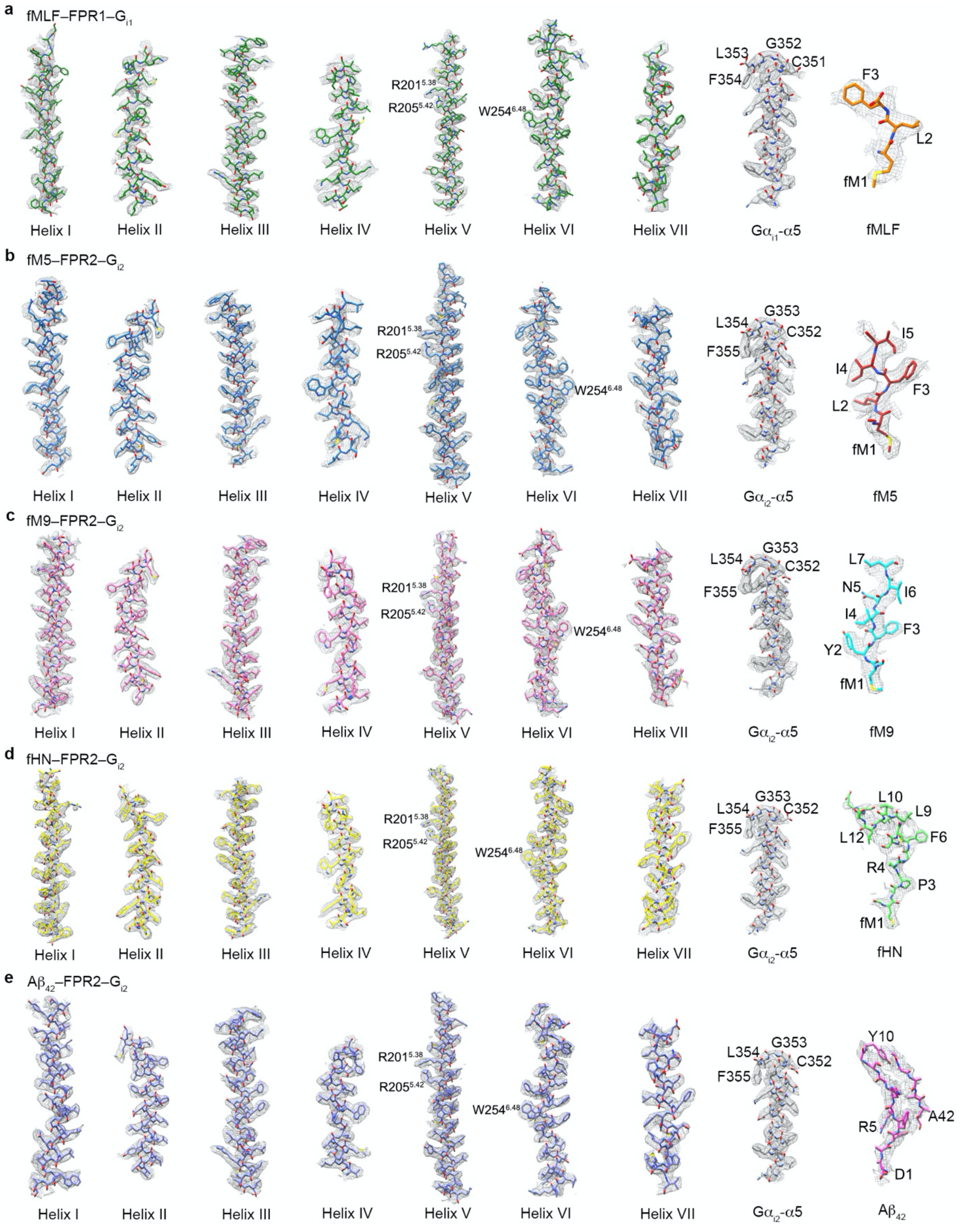

Supplementary Figure 3. Cryo-EM maps of the FPR1 and FPR2 structures. a, Cryo-EM density map and model of the fMLF–FPR1–G_{i1} structure are displayed for all receptor transmembrane helices, $G\alpha_{i1}$ α5-helix and fMLF. The structure model is shown as sticks with dark green (FPR1), gray ($G\alpha_{i1}$) and orange (fMLF) carbons. **b**, Cryo-EM density map and

model of the fM5–FPR2–G_{i2} structure are displayed for all receptor transmembrane helices, $G\alpha_{i2}$ α5-helix and fM5. The structure model is shown as sticks with blue (FPR2), gray ($G\alpha_{i2}$) and dark red (fM5) carbons. **c**, Cryo-EM density map and model of the fM9–FPR2–G_{i2} structure are displayed for all receptor transmembrane helices, $G\alpha_{i2}$ α5-helix and fM9 (fM1-L7). The structure model is shown as sticks with pink (FPR2), gray ($G\alpha_{i2}$) and cyan (fM9) carbons. **d**, Cryo-EM density map and model of the fHN–FPR2–G_{i2} structure are displayed for all receptor transmembrane helices, $G\alpha_{i2}$ α5-helix and fHN (fM1-E15). The structure model is shown as sticks with yellow (FPR2), gray ($G\alpha_{i2}$) and green (fHN) carbons. **e**, Cryo-EM density map and model of the $A\beta_{42}$–FPR2–G_{i2} structure are displayed for all receptor transmembrane helices, $G\alpha_{i2}$ α5-helix and $A\beta_{42}$ (D1-Y10 and G37-A42). The structure model is shown as sticks with blue (FPR2), gray ($G\alpha_{i2}$) and magenta ($A\beta_{42}$) carbons.

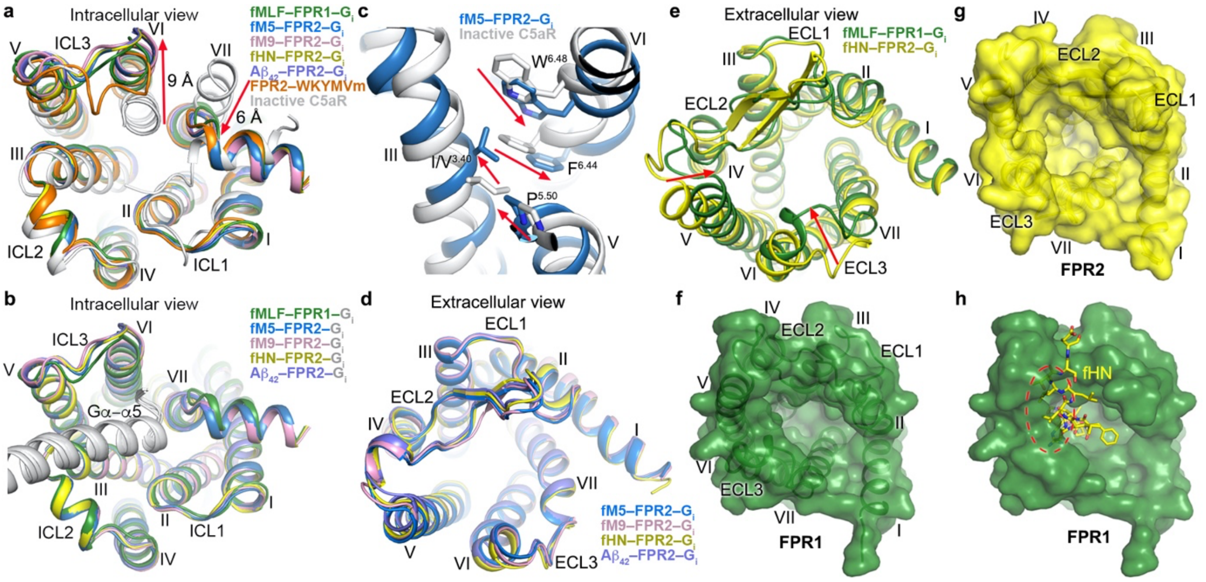

Supplementary Figure 4. Comparison of FPR1 and FPR2 structures. a, Comparison of the intracellular regions in the structures of fMLF–FPR1–G_{i1}, fM5–FPR2–G_{i2}, fM9–FPR2–G_{i2}, fHN–FPR2–G_{i2}, $A\beta_{42}$–FPR2–G_{i2}, FPR2–WKYMVm (PDB ID: 6LW5) and inactive C5aR (PDB ID: 6C1R). The red arrows indicate the movements of the intracellular tips of helices VI and VII in the active FPR structures relative to those in the inactive C5aR structure. **b**, Comparison of G protein binding in the G_i-bound FPR1 and FPR2 structures. The α5-helix in the Gα subunit is colored gray. **c**, Conformational comparison of the conserved 'toggle switch' and PIF motif in the structures of fM5–FPR2–G_{i2} and inactive C5aR. The key residues are shown as sticks. The red arrows indicate the movements of the key residues in the active FPR2 structure relative to those in the inactive C5aR structure. **d**, Structural comparison of the extracellular regions in the peptide agonist–FPR2–G_{i2} structures. **e**, Structural comparison of the extracellular regions in the structures of fMLF–FPR1–G_{i1} and fHN–FPR2–G_{i2}. The red arrows indicate the movements of ECL3 and the extracellular tip of helix V in the FPR2 structure relative to those in the FPR1 structure. **f**, **g**, Different opening of the ligand binding pockets in FPR1 and FPR2. The receptors in the structures of fMLF–FPR1–G_{i1} (**f**) and fHN–FPR2–G_{i2} (**g**) are shown in cartoon and surface representations. **h**, Superposition of the fMLF–FPR1–G_{i1} and fHN–FPR2–G_{i2} structures, showing the steric clash between FPR1 and fHN. The

receptor in the fMLF–FPR1–G_{i1} structure is shown as surface, and fHN in the fHN–FPR2–G_{i2} structure is shown as yellow sticks. The clashed region is highlighted by a red dashed circle.

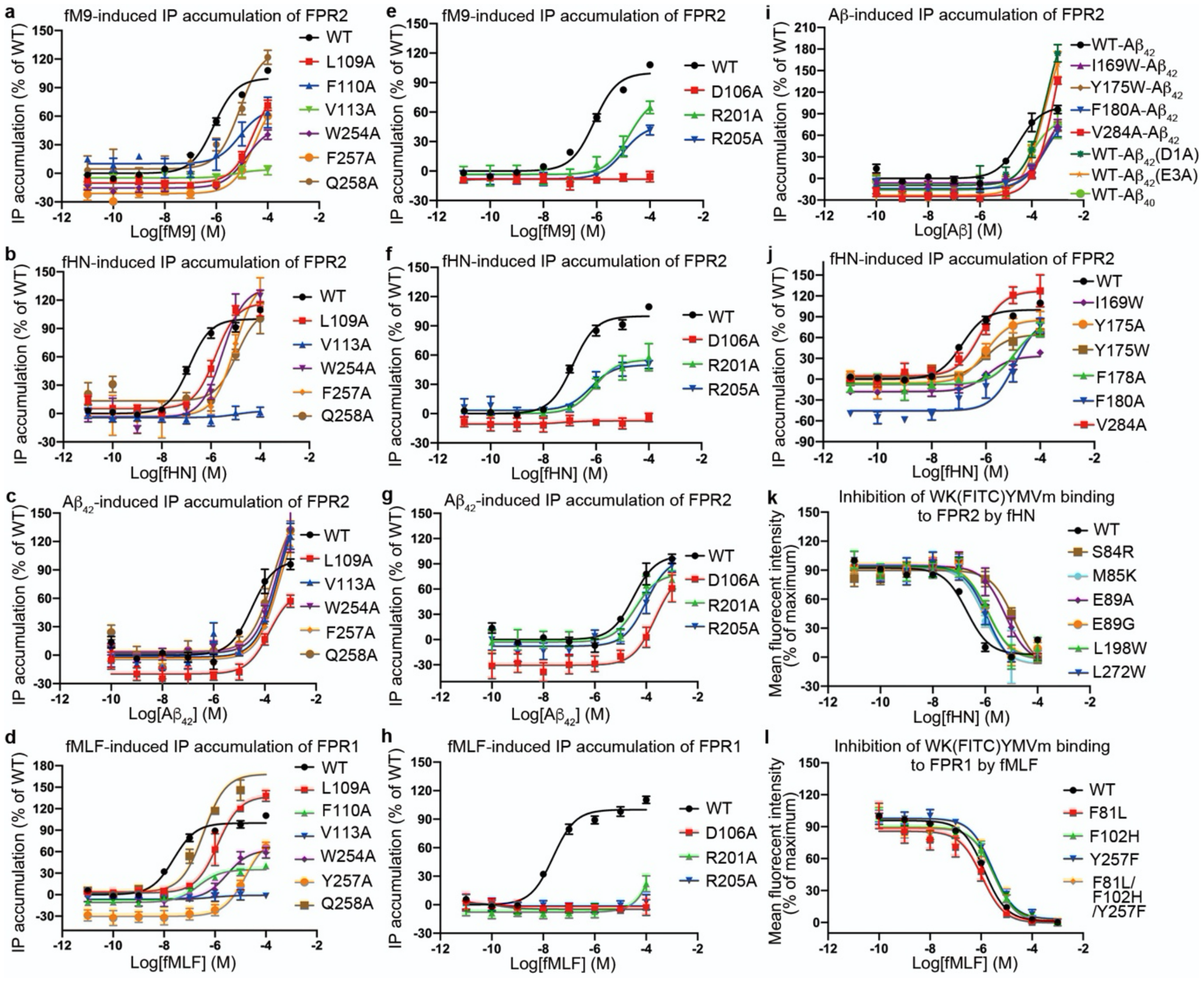

Supplementary Figure 5. Ligand binding and cell signalling assays of FPR1 and FPR2. Dose-response curves are generated from at least three independent experiments performed in triplicate. Data are shown as mean ± SEM. Supplementary Tables 1, 3 provide detailed numbers of independent experiments (n), statistical evaluation and expression levels. Source data are provided as a Source Data file. **a**, **e**, fM9-induced IP accumulation of wild-type FPR2 (WT) and mutants. **b**, **f**, **j**, fHN-induced IP accumulation of wild-type FPR2 and mutants. **c**, **g**, **i**, $A\beta_{42}$-induced IP accumulation of wild-type FPR2 and mutants. **d**, **h**, fMLF-induced IP accumulation of wild-type FPR1 (WT) and mutants. **k,** Inhibition of WK(FITC)YMVm binding to wild-type FPR2 and mutants by fHN. **l**, Inhibition of WK(FITC)YMVm binding to wild-type FPR1 and mutants by fMLF.

Supplementary Table 1. Binding of WK(FITC)YMVm to wild-type (WT) and mutant FPRs and inhibition by peptide agonists

Ligand binding assays of FPR1

FPR1 mutans	WK(FITC)YMVm, saturation binding			fMLF, competition binding					Surface expression[f]	
	K_d[a] (nM)	n[b]	Span[a,c] (% of WT)	K_i (μM)	K_i ratio[d]	pK_i mean ± SEM[a,e]	pK_i P value	n[b]	% of WT[e]	P value
WT	8.1 ± 1.2	8	100 ± 3	0.60	1	6.22 ± 0.03	/	20	100	/
Construct 1[g]	3.9 ± 1.7	3	31 ± 3	1.5	2	5.84 ± 0.10*	0.0260	3	89 ± 7	0.9958
F81$^{2.60}$L[h]	6.0 ± 4.3	3	60 ± 9	0.41	1	6.38 ± 0.18	0.6286	4	247 ± 6***	<0.0001
F102$^{3.29}$A	10 ± 3	3	152 ± 10	3.4	6	5.46 ± 0.10***	<0.0001	4	109 ± 17	0.9954
F102$^{3.29}$H	7.9 ± 5.0	3	135 ± 19	1.4	2	5.85 ± 0.11*	0.0138	4	300 ± 43***	<0.0001
Y257$^{6.51}$F	3.0 ± 1.2	3	61 ± 5	0.64	1	6.19 ± 0.09	0.9998	3	87 ± 7	0.9955
A264$^{6.58}$W	8.9 ± 3.8	3	102 ± 10	6.7	11	5.18 ± 0.16***	<0.0001	3	68 ± 10	0.6613
F81$^{2.60}$L/ F102$^{3.29}$H/ Y257$^{6.51}$F	9.9 ± 3.2	3	139 ± 10	1.7	3	5.76 ± 0.16*	0.0013	4	124 ± 18	0.8248

Ligand binding assays of FPR2

FPR2 mutants	WK(FITC)YMVm, saturation binding			fM9, competition binding					Surface expression[f]	
	K_d[a] (nM)	n[b]	Span[a,c] (% of WT)	K_i (μM)	K_i ratio[d]	pK_i mean ± SEM[a]	pK_i P value	n[b]	% of WT[e]	P value
WT	10 ± 1	18	100 ± 2	1.4	1	5.85 ± 0.04	/	18	100	/
Construct 2[g]	12 ± 2	4	103 ± 6	2.7	2	5.58 ± 0.12	/	4	149 ± 25***	<0.0001

FPR2 mutants	WK(FITC)YMVm, saturation binding			fHN, competition binding					Surface expression[f]	
	K_d[a] (nM)	n[b]	Span[a,c] (% of WT)	K_i (μM)	K_i ratio[d]	pK_i mean ± SEM[a,e]	pK_i P value	n[b]	% of WT[e]	P value
WT	10 ± 1	18	100 ± 2	0.10	1	7.00 ± 0.06	/	27	100	/
Construct 2[g]	12 ± 2	4	103 ± 6	0.53	5	6.28 ± 0.17*	0.0129	3	149 ± 25***	<0.0001
S84$^{2.63}$R[h]	6.6 ± 1.9	3	102 ± 8	4.9	49	5.31 ± 0.18***	<0.0001	4	57 ± 4**	0.0001
M85$^{2.64}$K	5.0 ± 3.3	3	42 ± 6	0.31	3	6.51 ± 0.26	0.0958	4	43 ± 3***	<0.0001
E89^{ECL1}A	6.4 ± 0.8	6	127 ± 4	2.2	22	5.65 ± 0.16***	<0.0001	3	77 ± 3	0.1108
E89^{ECL1}G	9.3 ± 3.2	3	87 ± 8	0.57	6	6.24 ± 0.13*	0.0020	4	142 ± 18***	<0.0001
L198$^{5.35}$A	19 ± 3	3	34 ± 2	nd	nd	nd	nd	3	78 ± 4	0.0758
L198$^{5.35}$W	9.1 ± 2.1	3	73 ± 4	0.65	7	6.19 ± 0.19**	0.0008	4	63 ± 3*	0.0010
L272^{ECL3}W	6.7 ± 1.9	4	42 ± 3	0.48	5	6.32 ± 0.24*	0.0063	4	59 ± 5**	0.0003

FPR2 mutans	WK(FITC)YMVm, saturation binding			Aβ42, competition binding					Surface expression[f]	
	K_d[a] (nM)	n[b]	Span[a,c] (% of WT)	K_i (μM)	K_i ratio[d]	pK_i mean ± SEM[a]	pK_i P value	n[b]	% of WT[e]	P value
WT	10 ± 1	18	100 ± 2	38	1	4.42 ± 0.04	/	18	100	/
Construct 2[g]	12 ± 2	4	103 ± 6	70	2	4.16 ± 0.08	/	3	149 ± 25***	<0.0001

fHN variants	fHN or fHN variant, competition binding				
	K_i (μM)	K_i ratio[d]	pK_i mean ± SEM[a,e]	pK_i P value	n[b]
WT	0.10	1	7.00 ± 0.06	/	27
M1A	20	200	4.70 ± 0.13***	<0.0001	4
A2W	0.05	0.5	7.33 ± 0.17	0.4087	3
P3A	0.81	8	6.09 ± 0.07***	<0.0001	3
R4A	0.28	3	6.55 ± 0.13	0.1031	3
F6A	0.10	1	7.00 ± 0.14	>0.9999	3
L11A	0.63	6	6.20 ± 0.07**	0.0005	3
L12A	0.82	8	6.08 ± 0.06***	<0.0001	3

[a]Data are shown as mean ± SEM from at least three independent experiments performed in triplicate. Source data are provided as a Source Data file.
[b]Sample size, the number of independent experiments performed in triplicate.
[c]The span is defined as the window between the maximal WKYMVm response (E_{max}) and the vehicle (no ligand). nd, not determined (data for which a robust concentration response curve could not be established within the concentration range tested, such that an E_{max} was not reached and therefore span could not be calculated).
[d]The K_i ratio ($K_{i(mutant)}/K_{i(WT)}$) represents the shift between the wild-type and mutant curves, and characterizes the effect of the mutations on ligand binding.
[e]$*P < 0.05$, $**P < 0.001$, $***P < 0.0001$ (one-way ANOVA followed by Dunnett's post-test, compared with the response of wild type).
[f]Protein expression levels of FPR1 and FPR2 constructs at the cell surface were determined in parallel by flow cytometry with an anti-FLAG antibody and reported as per cent compared to the wild type from at least three independent measurements performed in duplicate.
[g]Construct 1, the FPR1 construct used to determine the fMLF–FPR1–G_{i1} structure; construct 2, the FPR2 construct used to determine the peptide agonist–FPR2–G_{i2} structures.
[h]All mutations were introduced in the wild-type receptor.

Supplementary Table 2. Cryo-EM data collection, refinement and validation statistics

	**fMLF–FPR1–G_{i1} (EMDB-32858) (PDB 7WVU)**	**fM5–FPR2–G_{i2} (EMDB-32859) (PDB 7WVV)**	**fM9–FPR2–G_{i2} (EMDB-32860) (PDB 7WVW)**	**fHN–FPR2–G_{i2} (EMDB-32861) (PDB 7WVX)**	**Aβ_{42}–FPR2–G_{i2} (EMDB- 32862) (PDB 7WVY)**
**Data collection and processing**					
Magnification	81,000	81,000	81,000	81,000	81,000
Voltage (kV)	300	300	300	300	300
Electron exposure ($e^{–}$/Å^2)	70	70	70	70	70
Defocus range (μm)	–0.8 ~ –1.5	–0.8 ~ –1.5	–0.8 ~ –1.5	–0.8 ~ –1.5	–0.8 ~ –1.5
Pixel size (Å)	1.045	1.045	1.045	1.045	1.045
Symmetry imposed	C1	C1	C1	C1	C1
Initial particle images (no.)	6,063,502	5,637,810	2,379,340	9,511,741	6,020,047
Final particle images (no.)	1,299,041	1,216,356	422,636	1,398,841	1,094,657
Map resolution (Å)	3.3	2.9	3.1	2.8	3.0
FSC threshold	0.143	0.143	0.143	0.143	0.143
Map resolution range (Å)	2.0 – 5.0	2.0 – 5.0	2.0 – 5.0	2.0 – 5.0	2.0 – 5.0
**Refinement**					
Initial model used (PDB code)	6DDE, 6LW5	6DDE, 6LW5	6DDE, 6LW5	6DDE, 6LW5	6DDE, 6LW5
Model resolution (Å)	3.4	3.0	3.2	2.9	3.1
FSC threshold	0.5	0.5	0.5	0.5	0.5
Map sharpening *B* factor (Å^2)	–135	–97	–85	–101	–96
Model composition					
Non-hydrogen atoms	6,862	7,020	7,169	7,070	7,094
Protein residues	909	911	924	920	922
Ligand residues	3	5	7	15	16
B factors (Å^2)					
Receptor	78.5	54.8	65.2	58.1	55.4
Ligand	77.1	75.3	71.0	80.0	64.4
G_i	65.8	52.0	58.1	55.7	69.0
R.m.s. deviations					
Bond lengths (Å)	0.002	0.002	0.002	0.002	0.002
Bond angles (°)	0.451	0.428	0.480	0.475	0.501
Validation					
MolProbity score	1.52	1.45	1.49	1.48	1.47
Clashscore	8.22	7.21	9.14	8.38	8.85
Poor rotamers (%)	0.00	0.00	0.00	0.00	0.13
Ramachandran plot					
Favored (%)	97.65	97.77	98.46	97.90	98.13
Allowed (%)	2.35	2.23	1.54	2.10	1.87
Disallowed (%)	0.00	0.00	0.00	0.00	0.00

Supplementary Table 3. IP accumulation assays of wild-type (WT) and mutant FPRs using the chimeric Gα protein Gα$_{Δ6qi4myr}$.

fMLF-induced IP accumulation of FPR1 mutants

FPR1 mutants[a]	EC$_{50}$ (nM)	pEC$_{50}$		EC$_{50}$ ratio[c]	Span[d]		n[e]	Surface expression[f]	
		mean ± SEM[b]	*P* value		% of WT[b]	*P* value		% of WT[b]	*P* value
WT	24	7.62 ± 0.08	/	1	100 ± 3	/	15	100	/
D106$^{3.33}$A[g]	nd	nd	nd	nd	nd	nd	4	228 ± 26***	<0.0001
L109$^{3.36}$A	1,278	5.90 ± 0.12***	<0.0001	53	135 ± 7*	0.0017	3	78 ± 15	0.9023
F110$^{3.37}$A	222	6.65 ± 0.14**	0.0002	9	46 ± 3***	<0.0001	3	177 ± 39*	0.0060
V113$^{3.40}$A	nd	nd	nd	nd	nd	nd	3	154 ± 41	0.1007
R201$^{5.38}$A	nd	nd	nd	nd	nd	nd	4	96 ± 9	0.9998
R205$^{5.42}$A	nd	nd	nd	nd	nd	nd	4	123 ± 24	0.8563
W254$^{6.48}$A	2,950	5.53 ± 0.23***	<0.0001	123	69 ± 7*	0.0060	3	82 ± 10	0.9669
Y257$^{6.51}$A	15,900	4.80 ± 0.17***	<0.0001	660	112 ± 12	0.5959	3	161 ± 24*	0.0496
Q258$^{6.52}$A	356	6.45 ± 0.13***	<0.0001	10	164 ± 8***	<0.0001	3	159 ± 6*	0.0055

fM9-induced IP accumulation of FPR2 mutants

FPR2 mutants[a]	EC$_{50}$ (μM)	pEC$_{50}$		EC$_{50}$ ratio[c]	Span[d]		n[e]	Surface expression[f]	
		mean ± SEM[b]	*P* value		% of WT[b]	*P* value		% of WT[b]	*P* value
WT	0.81	6.09 ± 0.05	/	1	100 ± 2	/	33	100	/
D106$^{3.33}$A[g]	nd	nd	nd	nd	nd	nd	4	54 ± 5***	<0.0001
L109$^{3.36}$A	30	4.53 ± 0.12***	<0.0001	37	106 ± 9	0.9944	3	155 ± 14***	<0.0001
F110$^{3.37}$A	8.1	5.09 ± 0.39***	<0.0001	10	58 ± 13**	0.0006	3	143 ± 11**	0.0001
V113$^{3.40}$A	nd	nd	nd	nd	nd	nd	5	131 ± 10*	0.0029
R201$^{5.38}$A	17	4.78 ± 0.26***	<0.0001	21	79 ± 13	0.1229	4	44 ± 4***	<0.0001
R205$^{5.42}$A	10	5.00 ± 0.21***	<0.0001	12	55 ± 7***	<0.0001	4	28 ± 5***	<0.0001
W254$^{6.48}$A	15	4.84 ± 0.14***	<0.0001	19	64 ± 6*	0.0046	3	111 ± 11	0.9956
F257$^{6.51}$A	33	4.49 ± 0.22***	<0.0001	40	108 ± 17	0.9727	3	77 ± 7	0.5454
Q258$^{6.52}$A	8.2	5.09 ± 0.22***	<0.0001	10	125 ± 15	0.0907	3	43 ± 12***	<0.0001

fHN-induced IP accumulation of FPR2 mutants

FPR2 mutants[a]	EC$_{50}$ (μM)	pEC$_{50}$		EC$_{50}$ ratio[c]	Span[d]		n[e]	Surface expression[f]	
		mean ± SEM[b]	*P* value		% of WT[b]	*P* value		% of WT[b]	*P* value
WT	0.13	6.90 ± 0.07	/	1	100 ± 3	/	23	100	/
D106$^{3.33}$A	nd	nd	nd	nd	nd	nd	5	54 ± 5***	<0.0001
L109$^{3.36}$A	1.5	5.82 ± 0.10***	<0.0001	12	112 ± 5	0.9119	4	155 ± 14***	<0.0001
F110$^{3.37}$A	0.11	7.00 ± 0.27	0.9996	1	54 ± 6**	0.0003	3	143 ± 11**	0.0001
V113$^{3.40}$A	nd	nd	nd	nd	nd	nd	4	131 ± 10*	0.0029
V167^{ECL2}A	0.33	6.48 ± 0.20	0.6742	3	57 ± 5**	0.0006	3	36 ± 5***	<0.0001
I169^{ECL2}W	1.2	5.92 ± 0.19*	0.0010	9	51 ± 5***	<0.0001	3	40 ± 8***	<0.0001
Y175^{ECL2}A	1.0	6.00 ± 0.23**	0.0006	8	91 ± 11	0.9898	4	28 ± 4***	<0.0001
Y175^{ECL2}W	1.0	5.98 ± 0.23*	0.0026	8	62 ± 7*	0.0039	3	48 ± 9***	<0.0001
F178^{ECL2}A	11	4.95 ± 0.21***	<0.0001	85	86 ± 11	0.9135	3	27 ± 3***	<0.0001
N179^{ECL2}A	3.4	5.50 ± 0.22***	<0.0001	26	59 ± 6*	0.0014	3	32 ± 5***	<0.0001
F180^{ECL2}A	12	4.94 ± 0.22***	<0.0001	89	134 ± 18*	0.0128	3	57 ± 3**	0.0008
R201$^{5.38}$A	0.88	6.06 ± 0.24*	0.0015	7	55 ± 6***	<0.0001	4	44 ± 4***	<0.0001
R205$^{5.42}$A	0.62	6.21 ± 0.28*	0.0174	5	47 ± 6***	<0.0001	4	28 ± 5***	<0.0001
W254$^{6.48}$A	3.0	5.52 ± 0.15***	<0.0001	23	137 ± 10*	0.0055	3	111 ± 11	0.9956
F257$^{6.51}$A	9.3	5.03 ± 0.20***	<0.0001	72	145 ± 17**	0.0003	3	77 ± 7	0.5454
Q258$^{6.52}$A	1.2	5.94 ± 0.20*	0.0013	9	104 ± 11	0.9996	3	43 ± 12***	<0.0001
V284$^{7.35}$A	0.65	6.19 ± 0.16*	0.0125	5	123 ± 10	0.1374	4	92 ± 19	0.9991

Aβ$_{42}$-induced IP accumulation of FPR2 mutants

FPR2 mutants[a]	EC$_{50}$ (μM)	pEC$_{50}$		EC$_{50}$ ratio[c]	Span[d]		n[e]	Surface expression[f]	
		mean ± SEM[b]	*P* value		% of WT[b]	*P* value		% of WT[b]	*P* value
WT	31	4.50 ± 0.15	/	1	100 ± 7	/	12	100	/
D106$^{3.33}$A	224	3.65 ± 0.30*	0.0396	7	112 ± 22	0.9905	4	54 ± 5***	<0.0001
L109$^{3.36}$A	150	3.82 ± 0.22	0.1567	5	89 ± 12	0.9911	4	155 ± 14***	<0.0001
F110$^{3.37}$A	39	4.40 ± 0.21	0.9996	1	134 ± 14	0.4060	3	143 ± 11**	0.0001

V113[3.40]A	nd	nd	nd	nd	nd	nd	3	131 ± 10*	0.0029
V167[ECL2]A	102	4.00 ± 0.17	0.5832	3	83 ± 9	0.9552	3	36 ± 5***	<0.0001
I169[ECL2]W	nd	nd	nd	nd	nd	nd	3	40 ± 8***	<0.0001
Y175[ECL2]W	nd	nd	nd	nd	nd	nd	4	48 ± 9***	<0.0001
F180[ECL2]A	nd	nd	nd	nd	nd	nd	4	57 ± 3**	0.0008
R201[5.38]A	38	4.42 ± 0.28	0.9996	1	81 ± 11	0.8124	5	44 ± 4***	<0.0001
R205[5.42]A	100	4.00 ± 0.21	0.3048	3	105 ± 13	0.9996	6	28 ± 5***	<0.0001
W254[6.48]A	nd	nd	nd	nd	nd	nd	4	111 ± 11	0.9956
F257[6.51]A	nd	nd	nd	nd	nd	nd	3	77 ± 7	0.5454
Q258[6.52]A	203	3.69 ± 0.21	0.1121	7	154 ± 21*	0.0396	3	43 ± 12***	<0.0001
V284[7.35]A	nd	nd	nd	nd	nd	nd	3	92 ± 19	0.9991

Aβ-induced IP accumulation of wild-type FPR2

Aβ[a]	EC_{50} (μM)	pEC_{50}		EC_{50} ratio[c]	Span[d]		n[e]
		mean ± SEM[b]	*P* value		% of WT[b]	*P* value	
$A\beta_{42}$–WT	31	4.50 ± 0.15	/	1	100 ± 7	/	12
$A\beta_{42}$–D1A	nd	nd	nd	nd	nd	nd	3
$A\beta_{42}$–E3A	nd	nd	nd	nd	nd	nd	3
$A\beta_{40}$	93	4.03 ± 0.19	0.5481	3	100 ± 10	>0.9999	4

[a]The chimeric $G\alpha_{\Delta6qi4myr}$-mediated IP accumulation assays were performed in parallel with the measurement of the IP production using the cells only transfected with the receptor as a control. The $G\alpha_{\Delta6qi4myr}$-mediated IP accumulation was calculated by subtracting the portion of control-mediated IP production for the WT and all the mutants.

[b]Data are shown as mean ± SEM from at least three independent experiments performed in triplicate. $*P < 0.05$, $**P < 0.001$, $***P < 0.0001$ (one-way ANOVA followed by Dunnett's post-test, compared with the response of wild type). Source data are provided as a Source Data file.

[c]The EC_{50} ratio ($EC_{50(mutant)}/EC_{50(WT)}$) represents the shift between the wild-type and mutant curves, and characterizes the effect of the mutations on receptor signalling.

[d]The span is defined as the window between the maximal agonist response (E_{max}) and the vehicle (no ligand). nd, not determined (data for which a robust concentration response curve could not be established within the concentration range tested, such that an E_{max} was not reached and therefore span could not be calculated).

[e]Sample size; the number of independent experiments performed in triplicate.

[f]Protein expression levels of FPR1 and FPR2 constructs at the cell surface were determined in parallel by flow cytometry with an anti-FLAG antibody and reported as per cent compared to the wild type from at least three independent measurements performed in duplicate.

[g]All mutations were introduced in the wild-type receptor.

Supplementary Table 4. DNA and primer sequences of FPR1 and FPR2

<table>
<tr><td colspan="2">FPR1-WT DNA sequence</td></tr>
<tr><td colspan="2">ATGGAAACTAACTCCAGCCTGCCTACTAACATCAGCGGCGGTACTCCTGCTGTGAGCGCCGGTTACCTGTTCC
TGGACATCATCACCTACCTGGTGTTCGCCGTGACCTTCGTGCTGGGTGTGCTGGGTAACGGTCTCGTGATCTG
GGTGGCTGGTTTCCGCATGACCCACACTGTGACCACCATCTCCTACCTGAACCTGGCTGTGGCTGACTTCTGT
TTCACTAGCACCCTGCCCTTCTTCATGGTGCGTAAGGCTATGGGTGGTCACTGGCCTTTCGGTTGGTTCCTGT
GTAAGTTCGTGTTCACCATCGTGGACATCAACCTGTTCGGTAGCGTGTTCCTGATCGCCCTGATCGCTCTGGA
CCGCTGTGTTTGTGTGCTCCACCCTGTGTGGACCCAGAACCACCGTACAGTGAGCCTGGCCAAGAAGGTTAT
CATCGGTCCATGGGTTATGGCTCTGCTGCTGACCCTGCCTGTGATCATCCGTGTGACCACTGTTCCTGGCAAG
ACTGGCACAGTGGCTTGTACATTCAACTTCAGCCCATGGACAAACGACCCTAAGGAACGCATCAACGTGGCC
GTGGCCATGCTGACTGTTCGTGGTATCATCCGCTTCATCATCGGTTTCAGCGCTCCTATGAGCATCGTGGCTGT
GAGCTACGGTCTGATCGCCACTAAGATCCACAAGCAGGGCCTCATCAAGTCCAGCCGTCCTCTGCGTGTGCT
GTCCTTCGTGGCCGCCGCTTTCTTCCTGTGTTGGAGCCCATACCAGGTGGTCGCCCTGATCGCTACAGTGCGC
ATCCGCGAGCTGCTGCAGGGTATGTACAAGGAGATCGGCATCGCTGTGGACGTGACCTCCGCCCTGGCTTTC
TTCAACTCCTGTCTGAACCCTATGCTGTACGTTTTCATGGGTCAGGACTTCCGCGAACGTCTGATCCACGCCC
TGCCAGCCAGCCTGGAGCGTGCCCTGACCGAGGACAGCACCCAGACCTCCGACACCGCCACCAACAGCAC
CCTGCCTTCCGCTGAAGTGGAACTGCAGGCTAAGTAG</td></tr>
<tr><td>WT-F</td><td>GCCGGCGCGCCGGAAACTAACTCCAGCCTGCC</td></tr>
<tr><td>C-truncation-R</td><td>CAGGAATTCCTCCAGGCTGGCTGGCAGGGC</td></tr>
<tr><td>F81L-F</td><td>TTCACTAGCACCCTGCCCTTCCTGATGGTGCGTAAGGCTATGGGT</td></tr>
<tr><td>F81L-R</td><td>ACCCATAGCCTTACGCACCATCAGGAAGGGCAGGGTGCTAGTGAA</td></tr>
<tr><td>F102A-F</td><td>TGGTTCCTGTGTAAGTTCGTGGCAACCATCGTGGACATCAACCTG</td></tr>
<tr><td>F102A-R</td><td>CAGGTTGATGTCCACGATGGTTGCCACGAACTTACACAGGAACCA</td></tr>
<tr><td>F102H-F</td><td>TGGTTCCTGTGTAAGTTCGTGCACACCATCGTGGACATCAACCTG</td></tr>
<tr><td>F102H-R</td><td>CAGGTTGATGTCCACGATGGTGTGCACGAACTTACACAGGAACCA</td></tr>
<tr><td>D106A-F</td><td>AAGTTCGTGTTCACCATCGTGGCAATCAACCTGTTCGGTAGCGTG</td></tr>
<tr><td>D106A-R</td><td>CACGCTACCGAACAGGTTGATTGCCACGATGGTGAACACGAACTT</td></tr>
<tr><td>L109A-F</td><td>TTCACCATCGTGGACATCAACGCTTTCGGTAGCGTGTTCCTGATC</td></tr>
<tr><td>L109A-R</td><td>GATCAGGAACACGCTACCGAAAGCGTTGATGTCCACGATGGTGAA</td></tr>
<tr><td>F110A-F</td><td>ACCATCGTGGACATCAACCTGGCAGGTAGCGTGTTCCTGATCGCC</td></tr>
<tr><td>F110A-R</td><td>GGCGATCAGGAACACGCTACCTGCCAGGTTGATGTCCACGATGGT</td></tr>
<tr><td>V113A-F</td><td>GACATCAACCTGTTCGGTAGCGCTTTCCTGATCGCCCTGATCGCT</td></tr>
<tr><td>V113A-R</td><td>AGCGATCAGGGCGATCAGGAAAGCGCTACCGAACAGGTTGATGTC</td></tr>
<tr><td>R201A-F</td><td>GCCGTGGCCATGCTGACTGTTGCAGGTATCATCCGCTTCATCATC</td></tr>
<tr><td>R201A-R</td><td>GATGATGAAGCGGATGATACCTGCAACAGTCAGCATGGCCACGGC</td></tr>
<tr><td>R205A-F</td><td>CTGACTGTTCGTGGTATCATCGCCTTCATCATCGGTTTCAGCGCT</td></tr>
<tr><td>R205A-R</td><td>AGCGCTGAAACCGATGATGAAGGCGATGATACCACGAACAGTCAG</td></tr>
<tr><td>W254A-F</td><td>GCCGCCGCTTTCTTCCTGTGTGCCAGCCCATACCAGGTGGTCGCC</td></tr>
<tr><td>W254A-R</td><td>GGCGACCACCTGGTATGGGCTGGCACACAGGAAGAAAGCGGCGGC</td></tr>
<tr><td>A264W-F</td><td>TACCAGGTGGTCGCCCTGATCTGGACAGTGCGCATCCGCGAGCTG</td></tr>
<tr><td>A264W-R</td><td>CAGCTCGCGGATGCGCACTGTCCAGATCAGGGCGACCACCTGGTA</td></tr>
<tr><td>Y257A-F</td><td>TTCTTCCTGTGTTGGAGCCCAGCACAGGTGGTCGCCCTGATCGCT</td></tr>
<tr><td>Y257A-R</td><td>AGCGATCAGGGCGACCACCTGTGCTGGGCTCCAACACAGGAAGAA</td></tr>
<tr><td>Y257F-F</td><td>TTCTTCCTGTGTTGGAGCCCATTCCAGGTGGTCGCCCTGATCGCT</td></tr>
<tr><td>Y257F-R</td><td>AGCGATCAGGGCGACCACCTGGAATGGGCTCCAACACAGGAAGAA</td></tr>
<tr><td>Q258A-F</td><td>TTCCTGTGTTGGAGCCCATACGCAGTGGTCGCCCTGATCGCTACA</td></tr>
<tr><td>Q258A-R</td><td>TGTAGCGATCAGGGCGACCACTGCGTATGGGCTCCAACACAGGAA</td></tr>
<tr><td colspan="2">FPR2-WT DNA sequence</td></tr>
<tr><td colspan="2">GGCGCGCCATGGAAACCAACTTCTCAACCCCGCTGAACGAATACGAGGAGGTCTCATACGAATCCGCAGGC
TACACGGTGCTCCGCATCTTGCCCTTGGTGGTCCTGGGCGTCACCTTCGTTCTCGGAGTGTTGGGAAACGGT
CTGGTCATCTGGGTTGCCGGTTTCAGGATGACCAGAACTGTTACCACTATTTGCTACTTGAACCTGGCTCTCG
CCGACTTCTCCTTCACAGCTACGCTGCCTTTCCTCATCGTGAGCATGGCCATGGGAGAGAAGTGGCCCTTCG
GCTGGTTCTTGTGTAAACTGATCCACATTGTTGTGGACATCAACTTGTTCGGATCTGTCTTCCTGATCGGTTTC
ATTGCTCTCGATCGCTGCATTTGTGTGCTGCACCCAGTCTGGGCACAGAACCATCGTACAGTGTCACTGGCG
ATGAAGGTCATCGTCGGCCCATGGATTTTGGCACTGGTTCTCACATTGCCGGTGTTCCTGTTCCTCACAACGG
TCACGATCCCAAACGGAGATACCTACTGCACTTTCAACTTCGCCTCCTGGGGTGGCACTCCGGAGGAAAGGT
TGAAGGTGGCAATCACAATGCTGACGGCGAGGGGCATCATTAGATTCGTGATTGGATTCTCTCTGCCTATGTC
AATCGTCGCTATTTGCTACGGTCTCATCGCTGCCAAGATTCACAAGAAAGGCATGATCAAATCCAGCCGCCCT
CTCCGTGTCTTGACCGCAGTCGTTGCGAGTTTCTTCATCTGTTGGTTCCCCTTCCAACTGGTCGCTCTGCTCG
GAACTGTTTGGCTGAAGGAGATGCTCTTCTACGGCAAGTACAAAATCATTGACATCCTCGTGAACCCAACTT
CTTCATTGGCTTTCTTCAACTCTTGTTTGAACCCGATGCTGTACGTTTTCGTGGGCCAGGATTTCCGCGAACG
TCTCATCCATAGTTTGCCTACATCGCTGGAGAGAGCTCTCTCCGAAGACAGCGCACCAACCAACGATACTGC
AGCGAACAGTGCTTCGCCTCCCGCCGAGACGGAACTGCAAGCCATGGAATTC</td></tr>
<tr><td>bRIL-F</td><td>ATTGGCGCGCCGGCTGATCTGGAAGACAATTGGGAA</td></tr>
</table>

bRIL-FPR2-F	ATTGGCGCGCCGACCAACTTCTCAACCCCG
C-truncation-R	CAGGAATTCCGTCTCGGCGGGAGGCGAAGC
S84R-F	ACGCTGCCTTTCCTCATCGTGCGCATGGCCATGGGAGAGAAGTGG
S84R-R	CCACTTCTCTCCCATGGCCATGCGCACGATGAGGAAAGGCAGCGT
M85K-F	CTGCCTTTCCTCATCGTGAGCAAGGCCATGGGAGAGAAGTGGCCC
M85K-R	GGGCCACTTCTCTCCCATGGCCTTGCTCACGATGAGGAAAGGCAG
E89A-F	TCATCGTGAGCATGGCCATGGGAGCAAAGTGGCCCTTCGGCTGGT
E89A-R	ACCAGCCGAAGGGCCACTTTGCTCCCATGGCCATGCTCACGATGA
E89G-F	ATCGTGAGCATGGCCATGGGAGGCAAGTGGCCCTTCGGCTGGTTC
E89G-R	GAACCAGCCGAAGGGCCACTTGCCTCCCATGGCCATGCTCACGAT
D106A-F	TAAACTGATCCACATTGTTGTGGCCATCAACTTGTTCGGATCTGT
D106A-R	ACAGATCCGAACAAGTTGATGGCCACAACAATGTGGATCAGTTTA
L109A-F	CACATTGTTGTGGACATCAACGCCTTCGGATCTGTCTTCCTGATC
L109A-R	GATCAGGAAGACAGATCCGAAGGCGTTGATGTCCACAACAATGTG
F110A-F	CATTGTTGTGGACATCAACTTGGCAGGATCTGTCTTCCTGATCGG
F110A-R	CCGATCAGGAAGACAGATCCTGCCAAGTTGATGTCCACAACAATG
V113A-F	GACATCAACTTGTTCGGATCTGCTTTCCTGATCGGTTTCATTGCT
V113A-R	AGCAATGAAACCGATCAGGAAAGCAGATCCGAACAAGTTGATGTC
V167A-F	GTGTTCCTGTTCCTCACAACGGCTACGATCCCAAACGGAGATACC
V167A-R	GGTATCTCCGTTTGGGATCGTAGCCGTTGTGAGGAACAGGAACAC
I169W-F	CTGTTCCTCACAACGGTCACGTGGCCAAACGGAGATACCTACTGC
I169W-R	GCAGTAGGTATCTCCGTTTGGCCACGTGACCGTTGTGAGGAACAG
Y175A-F	ACGATCCCAAACGGAGATACCGCTTGCACTTTCAACTTCGCCTCC
Y175A-R	GGAGGCGAAGTTGAAAGTGCAAGCGGTATCTCCGTTTGGGATCGT
Y175W-F	ACGATCCCAAACGGAGATACCTGGTGCACTTTCAACTTCGCCTCC
Y175W-R	GGAGGCGAAGTTGAAAGTGCACCAGGTATCTCCGTTTGGGATCGT
F178A-F	AACGGAGATACCTACTGCACTGCAAACTTCGCCTCCTGGGGTGGC
F178A-R	GCCACCCCAGGAGGCGAAGTTTGCAGTGCAGTAGGTATCTCCGTT
N179A-F	GGAGATACCTACTGCACTTTCGCTTTCGCCTCCTGGGGTGGCACT
N179A-R	AGTGCCACCCCAGGAGGCGAAAGCGAAAGTGCAGTAGGTATCTCC
F180A-F	GATACCTACTGCACTTTCAACGCAGCCTCCTGGGGTGGCACTCCG
F180A-R	CGGAGTGCCACCCCAGGAGGCTGCGTTGAAAGTGCAGTAGGTATC
L198A-F	TTGAAGGTGGCAATCACAATGGCCACGGCGAGGGGCATCATTAGA
L198A-R	TCTAATGATGCCCCTCGCCGTGGCCATTGTGATTGCCACCTTCAA
L198W-F	TTGAAGGTGGCAATCACAATGTGGACGGCGAGGGGCATCATTAGA
L198W-R	TCTAATGATGCCCCTCGCCGTCCACATTGTGATTGCCACCTTCAA
R201A-F	GCAATCACAATGCTGACGGCGGCAGGCATCATTAGATTCGTGATT
R201A-R	AATCACGAATCTAATGATGCCTGCCGCCGTCAGCATTGTGATTGC
R205A-F	CTGACGGCGAGGGGCATCATTGCCTTCGTGATTGGATTCTCTCTG
R205A-R	CAGAGAGAATCCAATCACGAAGGCAATGATGCCCCTCGCCGTCAG
S211L-F	TAGATTCGTGATTGGATTCCTGCTGCCTATGTCAATCGTCGCTAT
S211L-R	ATAGCGACGATTGACATAGGCAGCAGGAATCCAATCACGAATCTA
W254A-F	GTTGCGAGTTTCTTCATCTGTGCCTTCCCCTTCCAACTGGTCGCT
W254A-R	AGCGACCAGTTGGAAGGGGAAGGCACAGATGAAGAAACTCGCAAC
F257A-F	GTTTCTTCATCTGTTGGTTCCCCGCCCAACTGGTCGCTCTGCTCG
F257A-R	CGAGCAGAGCGACCAGTTGGGCGGGGAACCAACAGATGAAGAAAC
Q258A-F	TTCATCTGTTGGTTCCCCTTCGCTCTGGTCGCTCTGCTCGGAACT
Q258A-R	AGTTCCGAGCAGAGCGACCAGAGCGAAGGGGAACCAACAGATGAA
L272W-F	ACTGTTTGGCTGAAGGAGATGTGGTTCTACGGCAAGTACAAAATC
L272W-R	GATTTTGTACTTGCCGTAGAACCACATCTCCTTCAGCCAAACAGT
V284A-F	TACAAAATCATTGACATCCTCGCTAACCCAACTTCTTCATTGGCT
V284A-R	AGCCAATGAAGAAGTTGGGTTAGCGAGGATGTCAATGATTTTGTA
